# TIGIT impairs NK cell antifibrotic activity through the IFNγ–IFI30 axis in schistosomiasis-induced liver fibrosis

**DOI:** 10.3389/fimmu.2026.1766930

**Published:** 2026-02-19

**Authors:** Hui Peng, Jing Zhang, Xiaocheng Zhang, Hao Zhou, Lijun Cui, Fangfang Xu, Tingting Jiang, Jianhai Yin, Yuan Hu, Yujuan Shen, Shaohong Lu, Jianping Cao

**Affiliations:** 1 National Key Laboratory of Intelligent Tracking and Forecasting for Infectious Diseases, National Institute of Parasitic Diseases at Chinese Center for Disease Control and Prevention (Chinese Center for Tropical Diseases Research), Shanghai, China; 2 Key Laboratory of Parasite and Vector Biology, National Health Commission of the People’s Republic of China, Shanghai, China; 3 World Health Organization Collaborating Centre for Tropical Diseases, Shanghai, China; 4 Engineering Research Center of Novel Vaccine of Zhejiang Province, Zhejiang Provincial Key Laboratory of High-level Biosafety and Biomedical Transformation, Hangzhou Medical College, Hangzhou, China; 5 The School of Global Health, Chinese Center for Tropical Diseases Research, Shanghai Jiao Tong University School of Medicine, Shanghai, China

**Keywords:** interferon gamma inducible protein 30 (IFI30), interferon-γ (IFN-γ), natural killer (NK), schistosomiasis, TIGIT

## Abstract

**Background:**

Schistosomiasis-induced liver fibrosis is a major cause of morbidity and mortality, driven largely by dysregulated immune responses. Natural killer (NK) cells are critical antifibrotic effectors; however, their functions are often impaired during chronic infection.

**Methods:**

To investigate the mechanism underlying NK cell dysfunction, we established a murine model of *Schistosoma japonicum* infection and assessed NK cell phenotype, cytokine production, and fibrosis markers. A *TIGIT*-knockdown NK92 cell line and NK cell–specific *Tigit*-deficient mice were generated to examine the regulatory role of TIGIT. RNA sequencing and functional assays were used to identify downstream effectors.

**Results:**

TIGIT expression on hepatic NK cells increased progressively during infection and was accompanied by reduced secretion of interferon-γ and granzyme B, indicating functional exhaustion. *TIGIT* knockdown restored NK cell cytotoxicity against hepatic stellate cells and upregulated interferon-induced transmembrane protein IFI30, enhancing NK cell proliferation through an interferon-γ–dependent mechanism. *In vivo*, NK cell–specific *TIGIT* deletion alleviated hepatic inflammation, collagen deposition, and fibrosis marker expression in schistosomiasis-infected mice.

**Conclusion:**

These findings identify TIGIT as a key negative regulator of NK cell antifibrotic activity and reveal an immunoregulatory TIGIT–interferon-γ–IFI30 axis that drives NK cell dysfunction and promotes schistosomiasis-induced liver fibrosis. Targeting this pathway may provide a new immunotherapeutic strategy for fibrotic diseases associated with chronic infection.

## Introduction

Schistosomiasis is a parasitic disease caused by infection with trematodes of the genus *Schistosoma*, primarily endemic to tropical and subtropical regions. According to World Health Organization statistics, schistosome transmission occurs in 78 countries and territories worldwide, affecting more than 240 million people each year and posing a serious threat to global health and socioeconomic development (1–3). *Schistosoma japonicum* remains the most prevalent species in China. Following infection, schistosome eggs become trapped in the liver and release soluble egg antigens, which trigger the activation and proliferation of hepatic stellate cells (HSCs). Activated HSCs transdifferentiate into myofibroblasts, leading to excessive extracellular matrix deposition and progressive liver fibrosis, the principal and most severe pathological feature in schistosomiasis patients (4–6). The 2024 National Surveillance and Control Report indicated that 86.22% of endemic areas in China have achieved elimination criteria; however, more than 10,000 schistosomiasis cases remain, of which 99.99% are in the advanced stage (7–9). Therefore, elucidating the mechanisms underlying schistosomiasis-induced liver fibrosis is critical for slowing disease progression and reducing the clinical burden among affected individuals.

Natural killer (NK) cells, key effector components of the innate immune system (10, 11), play an essential regulatory role in the development and resolution of liver fibrosis (12, 13). Previous studies have demonstrated that NK cell deficiency exacerbates fibrotic progression, whereas adoptive transfer of activated NK cells markedly attenuates fibrosis (14). NK cells exert their antifibrotic effects primarily by selectively eliminating activated HSCs (15). This cytotoxicity is mediated through activating receptors such as NKG2D, which recognize stress-induced ligands including MICA/B expressed on activated HSCs (16, 17). Engagement of these receptors triggers HSCs apoptosis via the perforin/granzyme and TRAIL–DR5 death receptor pathways (18). In parallel, activated NK cells secrete high levels of interferon-γ (IFN-γ), which directly induces apoptosis of HSCs and mitigates fibrotic progression (19, 20). Therefore, the balance between activating and inhibitory receptor signaling on NK cells is a critical determinant of their functional state, whether activated or exhausted, and consequently of their capacity to regulate liver fibrosis.

Our previous study demonstrated that NK cells are activated and exert anti-infective functions during the early phase of *S*. *japonicum* infection (2–4 weeks post-infection), whereas functional exhaustion becomes evident by 6 weeks post-infection (21). Transcriptomic analysis further revealed that the upregulation of the inhibitory receptor TIGIT on NK cells may represent a key mechanism underlying this exhaustion (22). As an important immune checkpoint molecule, TIGIT plays a pivotal role in modulating NK cell activity. Previous research has shown that TIGIT expression is markedly elevated on NK cells in chronic viral infections such as hepatitis C virus (HCV) and human immunodeficiency virus (HIV) infection, as well as in various tumor microenvironments, where it is closely associated with a functionally exhausted phenotype. Notably, blockade of TIGIT signaling has been shown to reverse NK cell dysfunction and enhance cytotoxic activity against hepatocellular carcinoma (HCC) cells (23, 24). These findings identify TIGIT as a critical negative regulator of NK cell function. Building upon these insights, the present study aims to elucidate the molecular mechanisms by which TIGIT upregulation drives NK cell exhaustion, thereby providing a theoretical foundation for the development of potential antifibrotic immunotherapeutic strategies.

## Materials and methods

### Ethics statement

All animal experiments were conducted in strict accordance with the Regulations for the Administration of Affairs Concerning Experimental Animals approved by the State Council of the People’s Republic of China and complied with internationally accepted guidelines for laboratory animal care and use. All procedures were designed to minimize animal suffering. Mice were humanely euthanized in accordance with the AVMA Guidelines for the Euthanasia of Animals (2020) using intraperitoneal injection of sodium pentobarbital. The study protocol was reviewed and approved by the Laboratory Animal Welfare & Ethics Committee (LAWEC) of the National Institute of Parasitic Diseases, Chinese Center for Disease Control and Prevention (Chinese Center for Tropical Diseases Research; approval ID: IPD-2022-015).

### Animals, cells and parasites

Six-week-old female C57BL/6 mice were obtained from Ji Hui Laboratory Animal Co., Ltd. (Shanghai, China). *Tigit*^fl/fl^-*Ncr1*^iCre/+^ mice were purchased from Mouse Treasure Biotechnology Co., Ltd. (Wuhan, China). *S. japonicum* cercariae (Chinese mainland strain) were provided by the snail breeding facility of our institute. All animals were maintained under specific pathogen-free (SPF) conditions in accordance with institutional animal care guidelines.

### Isolation and analysis of mice nonparenchymal hepatocytes

Female C57BL/6 mice were infected with *S. japonicum* cercariae using the abdominal patch method (20 ± 1 cercariae per mouse). Six mice were sacrificed prior to infection, and at 4, 6 and 8 weeks post-infection. All mice were humanely euthanized in strict accordance with the AVMA Guidelines for the Euthanasia of Animals (2020) via intraperitoneal injection of sodium pentobarbital (200 mg/kg body weight). Livers were perfused through the hepatic portal vein with phosphate-buffered saline (PBS). Nonparenchymal hepatic cells were isolated by double-density Percoll gradient centrifugation (Cytiva, Uppsala, Sweden). The white, cloud-like interphase fraction containing nonparenchymal cells was collected, washed twice with PBS, and treated with red blood cell lysis buffer to remove erythrocytes. The cell concentration was adjusted to 1 × 10^5^ cells/mL in fluorescence-activated cell sorting (FACS) buffer.

For flow cytometric analysis, cells were stained with the following fluorochrome-conjugated antibodies: Annexin V–BV605, CD3e–BB700, NK1.1–AF700, TIGIT–PE, IFNγ–PE–Cy7, and GZMB–APC (all from BD Biosciences, San Diego, CA, USA). NK cells were defined as CD3^−^NK1.1^+^ populations. Staining was performed for 30 min at room temperature (24–26 °C) and cells were washed once with FACS buffer. Data acquisition was performed using a BD FACSVerse flow cytometer (BD Biosciences), and data were analyzed with FlowJo v10 software (BD Biosciences). The proportions of NK cells and their intracellular levels of IFNγ and GZMB were subsequently evaluated.

### Establishment of *TIGIT*-knockdown NK92 cell and cell cycle detection

The human NK92 cell line was obtained from the National Collection of Authenticated Cell Cultures (Shanghai, China). Cells were maintained in MyeloCult™ H5100 medium (StemCell Technologies, Vancouver, Canada) supplemented with 12.6% horse serum (Gibco, Grand Island, NY, USA) and 100 U/mL recombinant human interleukin-2 (IL-2; Miltenyi Biotec, Bergisch Gladbach, Germany). Cultures were incubated at 37 °C in a humidified atmosphere containing 5% CO_2_.

To establish *TIGIT*-knockdown NK92 cells, the lentiviral vector pLent-EF1α-H1-GFP-Puro carrying the *TIGIT* short hairpin RNA (shRNA) sequence was used. The control and *TIGIT* -targeting lentiviruses were packaged and synthesized by Angel Biotechnology Co., Ltd. (Suzhou, China). NK92 cells were transduced with either the control or *TIGIT* knockdown lentivirus at a multiplicity of infection (MOI) of 100. After 72 h, puromycin (2 µg/mL) was added to the culture medium for selection, and stable cell lines were obtained after two weeks of continuous screening. The resulting negative control (NC) and *TIGIT*-knockdown (KD) NK92 cell lines were maintained under identical conditions.

For cell cycle analysis, cells were stained using a Cell Cycle Detection Kit (Beyotime Biotechnology Co., Ltd., Shanghai, China) according to the manufacturer’s protocol, followed by flow cytometric analysis. Data were acquired using a BD FACSVerse flow cytometer (BD Biosciences, San Diego, CA, USA) and analyzed with FlowJo v10 software (BD Biosciences) to determine the distribution of cells across different phases of the cell cycle.

### Detection of NK cell function

The human hepatic stellate cell line LX-2 was obtained from the Shanghai Cell Bank of the Chinese Academy of Sciences (Shanghai, China) and cultured in Dulbecco’s Modified Eagle’s Medium (DMEM; Gibco, Grand Island, NY, USA) supplemented with 10% fetal bovine serum (FBS; Gibco) at 37 °C in a humidified atmosphere containing 5% CO_2_. NK92 cells from the negative control (NC) and *TIGIT*-knockdown (KD) groups were co-cultured with LX-2 cells at a ratio of 5:1 for 12 h.

After co-culture, LX-2 cells were harvested, and apoptosis was evaluated using an Annexin V/PI Apoptosis Detection Kit (Elabscience Biotechnology Co., Ltd., Wuhan, China) according to the manufacturer’s instructions. Flow cytometric analysis was performed on a BD FACSVerse flow cytometer (BD Biosciences, San Diego, CA, USA), and data were analyzed with FlowJo v10 software (BD Biosciences) to determine the proportion of apoptotic cells.

In parallel, the expression levels of Gzmb and Prf1 in NK92 cells were analyzed by flow cytometry and quantitative reverse transcription PCR (RT-qPCR). For flow cytometry, cells were stained with GZMB–PE and PRF1–PE–Cy7 antibodies (Thermo Fisher Scientific, Waltham, MA, USA) following standard protocols.

### Treatment of NK92 cells with IFNγ antagonists

NK92 cells were seeded in six-well plates at a density of 1 × 10^6^ cells/mL and treated with 100 μmol IFNγ antagonist 1 (MedChemExpress, Monmouth Junction, NJ, USA) for 48 h. Cells cultured under identical conditions without antagonist treatment served as the control group.

### RNA extraction, reverse transcription and RT-qPCR analysis

Total RNA was extracted from mouse liver tissue and NK92 cells using a Cell and Tissue RNA Extraction Kit (Sikejie Biotechnology Co., Ltd., Shandong, China) according to the manufacturer’s instructions. The concentration and purity of RNA were determined spectrophotometrically, and 1 μg of total RNA was reverse-transcribed into complementary DNA (cDNA) using a Reverse Transcription Kit (Sikejie Biotechnology). Quantitative real-time PCR (RT-qPCR) was then performed to evaluate the expression of target genes associated with liver fibrosis (*Acta2*, *Col1a1*, *Fn*) and NK cell function (*Tigit*, *Ifnγ*, *Gzmb*, *Ifi30*). Primer sequences used in this study are listed in **Table 1**.

**Table 1:** Primer sequences for qPCR.

Gene name	Forward primer sequence (5'→3')	Reverse primer sequences (5'→3')
*GAPDH* c	AAGGTGAAGGTCGGAGTCAAC	GGGGTCATTGATGGCAACAATA
*Gapdh* (Mouse)	TGTTCCTACCCCCAATGTGTC	TGAAGTCGCAGGAGACAACC
*TIGIT* (Human)	TGGTGGTCATCTGCACAGCAGT	TTTCTCCTGAGGTCACCTTCCAC
*Tigit* (Mouse)	GCTGTGCTGGGACTCATTTG	GAGAGACTCCTCAGGTTCCATTC
*GZMB* (Human)	GTGGCTTCCTGATACGAGACG	AACTGCTGGGTCGGCTCC
*Gzmb* (Mouse)	TCCCCGTCTCTTCGTAAGC	AGGAGCTGTCAGACCCCTTC
*IFNG* (Human)	TGTGGAGACCATCAAGGAAGAC	ATGTATTGCTTTGCGTTGGAC
*Ifnγ* (Mouse)	CAGCAACAGCAAGGCGAAAAAGG	TTTCCGCTTCCTGAGGCTGGAT
*Acta2*	CCCTGAAGAGCATCCGACA	CTCCAGAGTCCAGCACAATACC
*Col1a1*	GAGGGCGAGTGCTGTGCT	GTCCAGGGATGCCATCTCG
*IFI30* (Human)	TGTCACGCTGGTGCCCTAC	CCTTGTTGAATTTGCACTCCTC
*Ifi30* (*Mouse*)	ATCCTCCGAAGGCACAACC	GGACGAGGAAGTAGCGACAAG
*SPRED3* (Human)	GTTTACAACAAGGTGAATCCCATC	TCTGAAACGTCAGTCCAAACTTG
*TREX2* (Human)	GAGCTGTCCCTCTTTGCTGTCC	GGCAATACTAGGGCACCAGACTC
*GNPDA1* (Human)	TGACATCCACCCAGAAAACACC	ACAAATAGCTCGATCCCACCTG
*IRAK2* (Human)	ACTTCAGCACCTCCATTCCTAAG	TGGCTGATTTTGCGGTTTTG
*TENM1* (Human)	TTGATCATATAACCCGCACAGG	ATCCCGAAGGTGAATATGTGATG
*SNX13* (Human)	AGACTGGCAACCTTATTTTACTACAC	CTGTTATTTTCTGTTGAGCCTTTC
*DAAM1* (Human)	GGACCTCACAGACAAACACAGAG	TTTCTTGCTACAGTATATTTGCCA
*SERPINE1* (Human)	CTCATCAGCCACTGGAAAGGCA	GACTCGTGAAGTCAGCCTGAAAC
*TNFSF13B* (Human)	AATCTTTGAACCACCAGCTCCA	GTTTCTTCTGGACCCTGAACGG

### Transcriptome analysis by RNA sequencing

NK92 cells from the NC and KD groups (three biological replicates per group) were collected and washed three times with PBS to remove residual contaminants. Total RNA was extracted using TRIzol Reagent (Invitrogen, Carlsbad, CA, USA) and quantified spectrophotometrically to assess purity and concentration. High-quality RNA samples were submitted to Biomarker Technologies Co., Ltd. (Beijing, China) for library preparation and sequencing. Differentially expressed genes (DEGs) were identified between the NC and KD groups. Functional enrichment and signaling pathway analyses of DEGs were performed using the Gene Ontology (GO) and Kyoto Encyclopedia of Genes and Genomes (KEGG) databases to elucidate the biological processes and molecular pathways affected by *TIGIT* knockdown.

### Hematoxylin and eosin and Masson staining

Liver tissues were fixed in 4% paraformaldehyde, routinely dehydrated, and paraffin-embedded. Tissue sections (4 µm thick) were stained using Hematoxylin and Eosin (HE) and Masson Staining Kits (Solarbio Science & Technology Co., Ltd., Beijing, China) according to the manufacturer’s protocols. For each group, three ± two representative sections containing single egg granulomas were imaged under a microscope using CellSens imaging software (Olympus, Tokyo, Japan). The proportion of positively stained areas was quantified using ImageJ software (National Institutes of Health, Bethesda, MD, USA).

### Quantification of liver egg burden

The hepatic egg burden was quantified using an alkaline digestion method to assess the intensity of *S. japonicum* infection. Mice were sacrificed at 6 weeks post-infection and humanely euthanized in strict accordance with the AVMA Guidelines for the Euthanasia of Animals (2020) via intraperitoneal injection of sodium pentobarbital (200 mg/kg body weight). The liver was immediately harvested, and approximately 0.1 g of tissue from each mouse was accurately weighed and transferred to a centrifuge tube. Liver samples were digested in 8 mL of 4% potassium hydroxide (KOH) solution at 37 °C for 2 h with constant shaking at 150 rpm to completely dissolve the tissue and release schistosome eggs. Following digestion, the suspension was thoroughly vortexed, and a 10 μL aliquot of the homogenized solution was placed onto a glass slide for egg counting under a light microscope. Egg counts were performed independently in triplicate for each sample, and the mean value was calculated. The total egg number in the entire digest was extrapolated based on the aliquot-to-total volume ratio. Hepatic egg burden was ultimately expressed as the number of eggs per gram of liver tissue.

### Western blot analysis

Total protein was extracted from liver tissue or cultured cells using 1 mL of RIPA lysis buffer supplemented with protease and phosphatase inhibitors (Solarbio Science & Technology Co., Ltd., Beijing, China). Lysates were centrifuged at 12,000 rpm (12,830 × g) for 5 min at 4 °C, and the protein concentration of the supernatant was determined using the bicinchoninic acid (BCA) assay (Solarbio Science & Technology). Equal amounts of protein (30–50 μg) were separated by sodium dodecyl sulfate–polyacrylamide gel electrophoresis (SDS–PAGE) and transferred onto polyvinylidene fluoride (PVDF) membranes using a semi-dry transfer apparatus (Bio-Rad, Hercules, CA, USA).

Membranes were blocked and then incubated overnight at 4 °C with the following primary antibodies: Mouse Anti-GAPDH (Cat. No. 5174; CST, Danvers, MA, USA), Rabbit Anti-Bcl-2 (Cat. No. 4223; CST), Rabbit Anti-Bax (Cat. No. 50599-2-Ig; ProteinTech, Wuhan, China), Rabbit Anti-Collagen I (Cat. No. 72026; CST), Rabbit Anti-α-Smooth Muscle Actin (α-SMA; Cat. No. 19245; CST), Rabbit Anti-TIGIT (Cat. No. 99568; CST), and Rabbit Anti-IFI30 (Cat. No. 15112-1-AP; ProteinTech). After washing, membranes were incubated with horseradish peroxidase (HRP)-conjugated secondary antibodies, sheep anti-rabbit IgG or sheep anti-mouse IgG (1:4000; Jackson ImmunoResearch, West Grove, PA, USA), for 1 h at room temperature. Immunoreactive bands were visualized using enhanced chemiluminescence (ECL) reagents (Solarbio Science & Technology).

Three to four biological replicates were included for each experimental group. Band intensities were quantified using ImageJ software (NIH, Bethesda, MD, USA), and protein expression levels were normalized to GAPDH.

### Statistical analysis

All statistical analyses were performed using GraphPad Prism 5.0 (GraphPad Software, San Diego, CA, USA) and Microsoft Excel 2010 (Microsoft, Redmond, WA, USA). Differences between two groups were evaluated using a two-tailed paired Student’s t-test or a two-tailed Mann–Whitney U test, as appropriate. Comparisons among multiple groups were conducted using one-way analysis of variance (ANOVA). Data are presented as mean ± standard deviation (SD), and a *P* value of less than 0.05 was considered statistically significant.

## Results

### High TIGIT expression is associated with NK cell exhaustion

**Figure 1:**
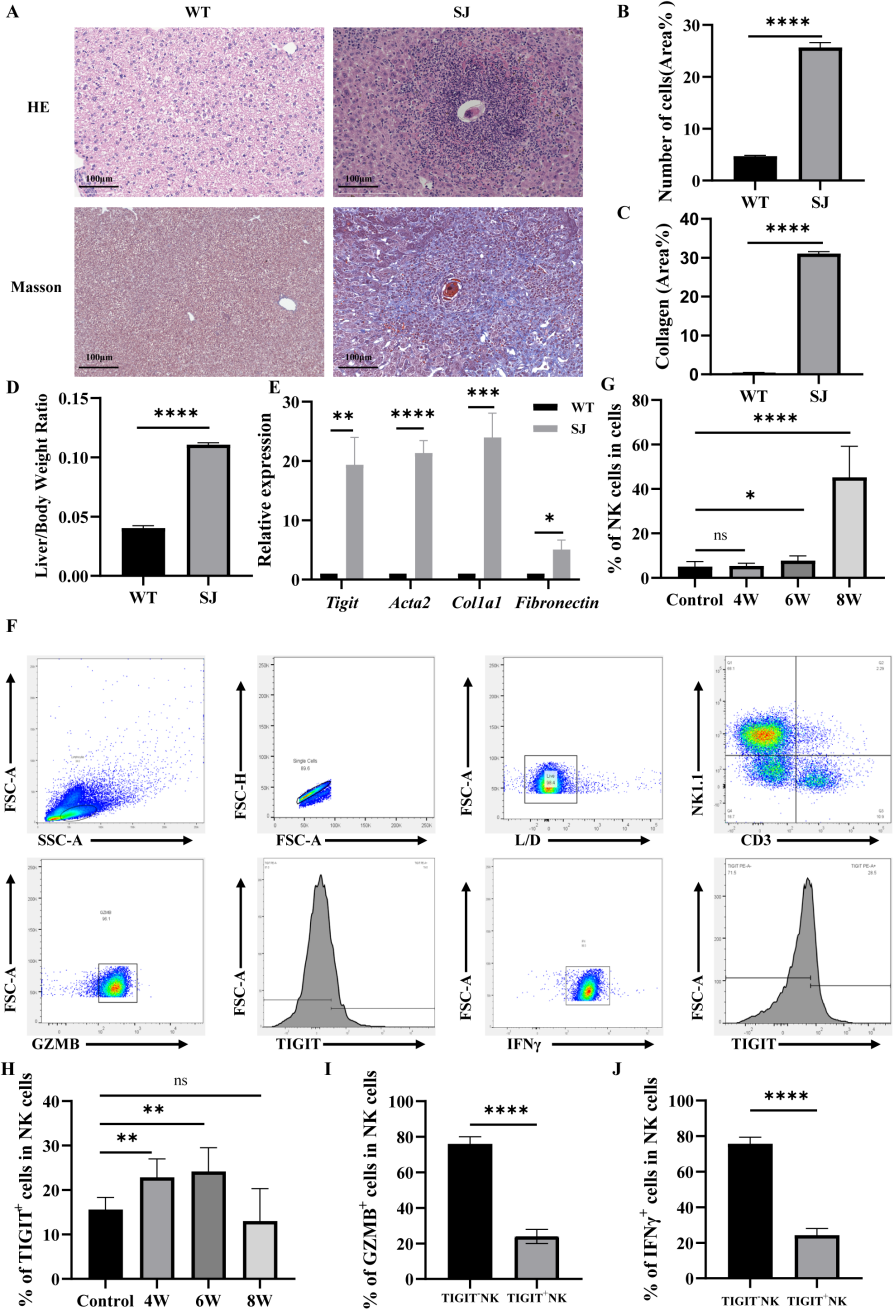
TIGIT upregulation on NK cells impairs their antifibrotic function during schistosomiasis infection. **(A)** Representative HE and Masson staining of liver tissue from control (WT)and *S. japonicum*–infected (SJ) mice at 6 weeks post-infection showing egg deposition and collagen accumulation. **(B)** Quantification of granulomatous inflammatory cell infiltration. **(C)** Quantification of collagen fiber deposition. **(D)** Liver-to-body weight ratio in control and infected mice. **(E)** Relative hepatic mRNA expression levels of *Acta2*, *Col I*, *Fn*, and *Tigit* at different time points post-infection. **(F)** Flow cytometric gating strategy for NK cells (CD3^−^NK1.1^+^) from hepatic nonparenchymal cells. **(G)** Proportion of NK cells at 0, 4, 6, and 8 weeks post-infection. **(H)** Percentage of TIGIT^+^ NK cells during infection. **(I, J)** Percentages of IFNγ^+^ and GZMB^+^ NK cells at different infection stages. *P < 0.05, **P < 0.01, ***P < 0.001, ****P < 0.0001.

A schistosomiasis infection mouse model was established by percutaneous exposure of mice to *S. japonicum cercariae*. HE and Masson staining confirmed the successful establishment of liver fibrosis at 6 weeks post-infection, as indicated by marked deposition of schistosome eggs within the liver (**Figure 1A**). Pronounced infiltration of inflammatory cells forming ring-shaped granulomas around the eggs and a substantial increase in collagen fiber accumulation were evident in these regions (**Figures 1B, C**). Consistently, the liver-to-body weight ratio was significantly elevated at 6 weeks post-infection, further validating the successful construction of the fibrosis model (**Figure 1D**). RT-qPCR analysis revealed that the expression levels of *Acta2*, *Col1a1*, and *Fn* were markedly upregulated in the liver at 6 weeks post-infection, accompanied by a significant increase in *Tigit* expression (**Figure 1E**).

Flow cytometric analysis of hepatic immune cells demonstrated a time-dependent dysregulation of NK cell populations during *S. japonicum* infection. Although the overall proportion of NK cells among hepatic nonparenchymal cells increased at 6 weeks post-infection (**Figures 1F, G**), there was a concomitant and progressive expansion of NK cells expressing the inhibitory immune checkpoint receptor TIGIT, which peaked at the same time point (**Figure 1H**). Importantly, these TIGIT^+^ NK cells displayed profound functional impairment, as indicated by significantly reduced production of IFNγ and granzyme B (**Figures 1I, J**). Our findings indicate that the progression of schistosomiasis-induced liver fibrosis is associated with the accumulation of TIGIT^+^ hepatic NK cells that exhibit functional impairment, defining an exhausted phenotype.

### *TIGIT* knockout upregulates IFN-γ expression and partially restores NK cell cytotoxic function

**Figure 2:**
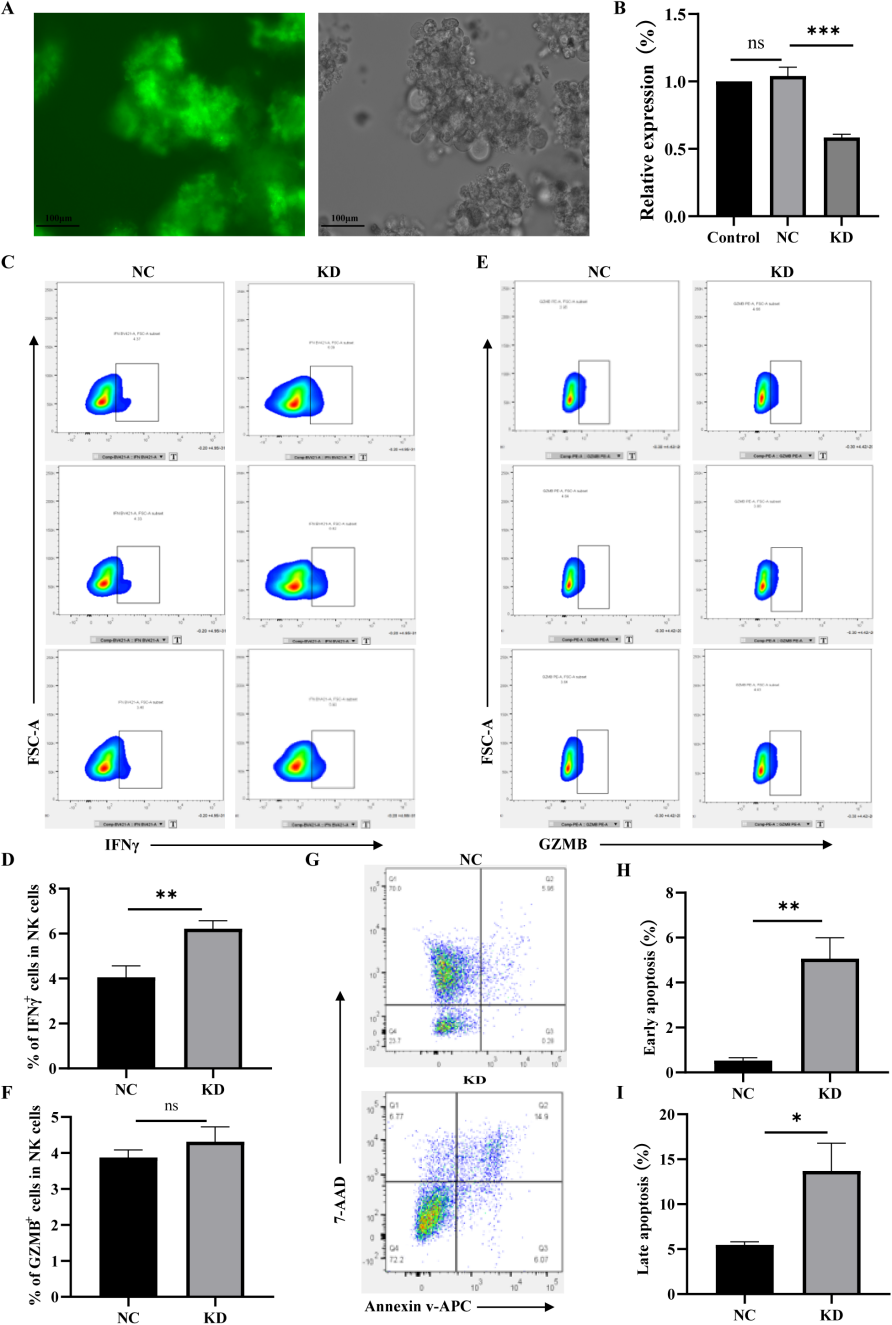
*TIGIT* knockdown enhances IFNγ production and partially restores NK92 cell cytotoxicity. **(A)** GFP fluorescence confirming successful lentiviral transduction of *TIGIT*-knockdown (KD) NK92 cells. **(B)** RT-qPCR verification of *TIGIT* knockdown efficiency. **(C, D)** Representative flow cytometry plots of IFNγ and GZMB expression in control (NC) and *TIGIT*-KD (KD) NK92 cells. **(E, F)** Quantification of IFNγ^+^ and GZMB^+^ cell proportions. **(G–I)** Flow cytometric analysis of apoptosis in LX-2 cells after co-culture with NC or *TIGIT*-KD NK92 cells for 6 h, showing increased early and late apoptosis induced by *TIGIT* knockdown. *P < 0.05, **P < 0.01, ***P < 0.001.

To investigate the effect of TIGIT on NK cell function, a *TIGIT*-knockdown NK92 cell line was established. As shown in **Figure 2A**, the transduced cells exhibited strong green fluorescence, confirming successful lentiviral transfection. RT-qPCR analysis further verified efficient *TIGIT* knockdown in NK92 cells (**Figure 2B**). Flow cytometric analysis demonstrated that *TIGIT* knockdown significantly increased IFNγ expression compared with control NK92 cells (**Figures 2C, E**), whereas GZMB expression remained unchanged (**Figures 2D, F**). Moreover, when co-cultured with LX-2 cells for 6 h, *TIGIT*-knockdown NK92 cells induced markedly higher levels of both early and late apoptosis in LX-2 cells compared with the control group (**Figures 2G–I**). These findings indicate that loss of TIGIT enhances IFNγ secretion and partially restores the cytotoxic activity of NK92 cells against hepatic stellate cells.

### RNA-seq reveals that *TIGIT* downregulation significantly alters IFI30 expression and affects NK cell activation

**Figure 3:**
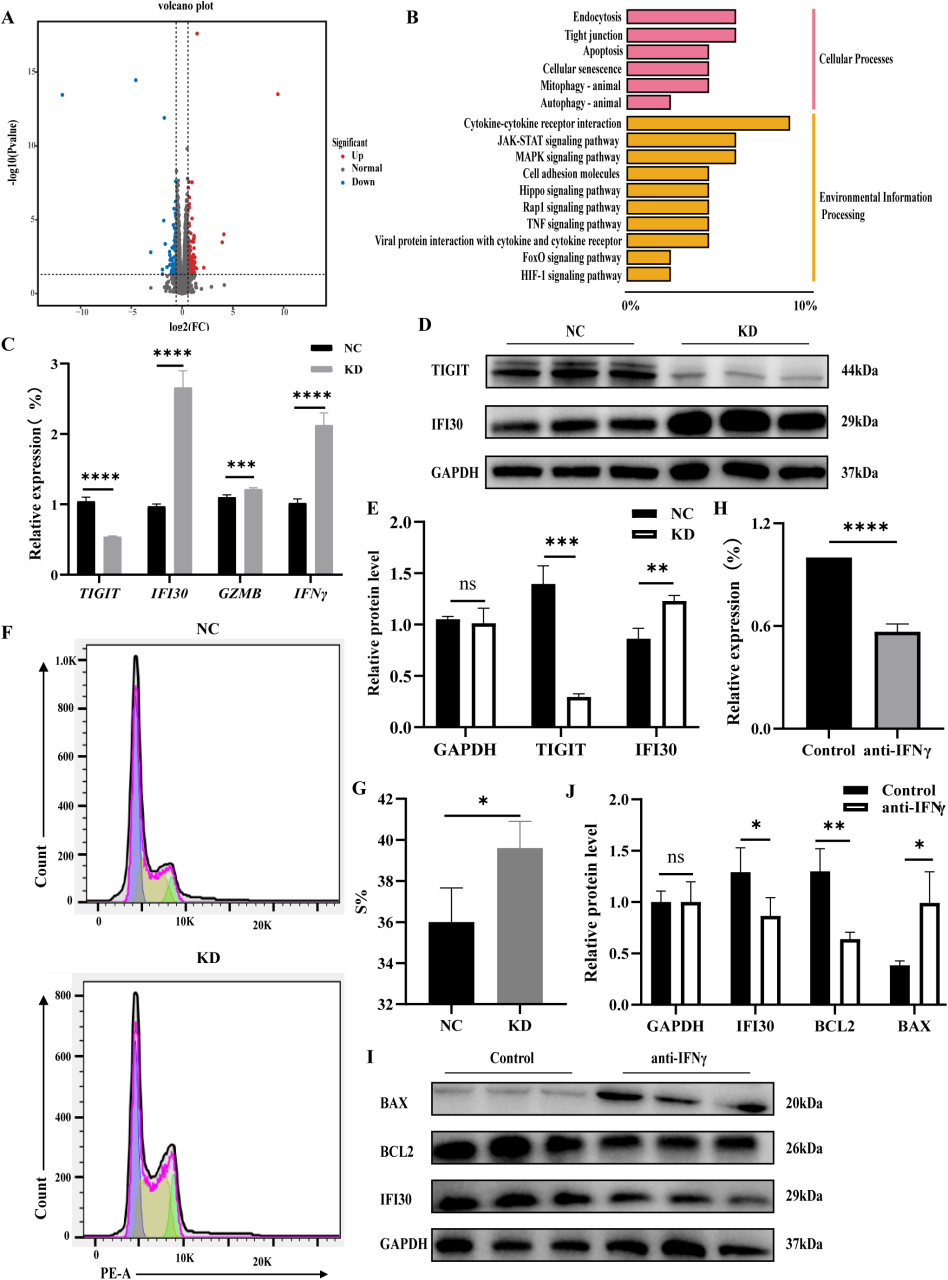
TIGIT downregulation alters IFI30 expression and promotes NK cell activation. **(A)** Volcano plot of differentially expressed genes. **(B)** KEGG pathway enrichment analysis of upregulated genes showing enrichment in cytokine and apoptotic signaling pathways. **(C–E)** Validation of *Ifi30*, *Gzmb*, and *Ifnγ* expression by RT-qPCR and WB, confirming IFI30 upregulation following *Tigit* knockdown. **(F, G)** Flow cytometric analysis of cell cycle distribution and quantification of S-phase proportion, indicating enhanced proliferation after *Tigit* knockdown. **(H–J)** RT-qPCR and WB analyses showing that IFNγ antagonist treatment downregulates Ifi30 and Bcl2 while increasing Bax expression, suggesting that the IFNγ–IFI30 axis regulates NK cell proliferation and apoptosis. *P < 0.05, **P < 0.01, ***P < 0.001, ****P < 0.0001.

RNA-seq analysis was performed to compare gene expression profiles between *TIGIT*-knockdown and control NK92 cells, with transcriptome validation and GO enrichment results shown in **Supplementary Figures 2A, B**. The resulting volcano plot of differentially expressed genes (DEGs) identified 122 upregulated and 84 downregulated genes following *TIGIT* knockdown (**Figure 3A**). KEGG pathway enrichment analysis revealed that the DEGs were primarily associated with immune cytokine signaling and apoptotic pathways (**Figure 3B**). Validation by RT-qPCR and WB confirmed that TIGIT downregulation led to a marked increase in IFI30 expression (**Figures 3C–E**). Flow cytometric analysis further showed that the proportion of NK92 cells in the proliferative (S) phase was significantly higher after *TIGIT* knockdown, indicating enhanced proliferative activity (**Figures 3F, G**). Treatment of NK92 cells with an IFNγ antagonist for 48 h resulted in downregulation of Ifi30 expression, accompanied by decreased expression of the anti-apoptotic protein BCL2 and increased expression of the pro-apoptotic protein BAX (**Figures 3H–J**). These findings suggest that TIGIT modulates NK cell proliferation and survival through the IFNγ–IFI30 signaling axis.

### NK-specific knockout of *TIGIT* ameliorates schistosomiasis-induced liver fibrosis

**Figure 4:**
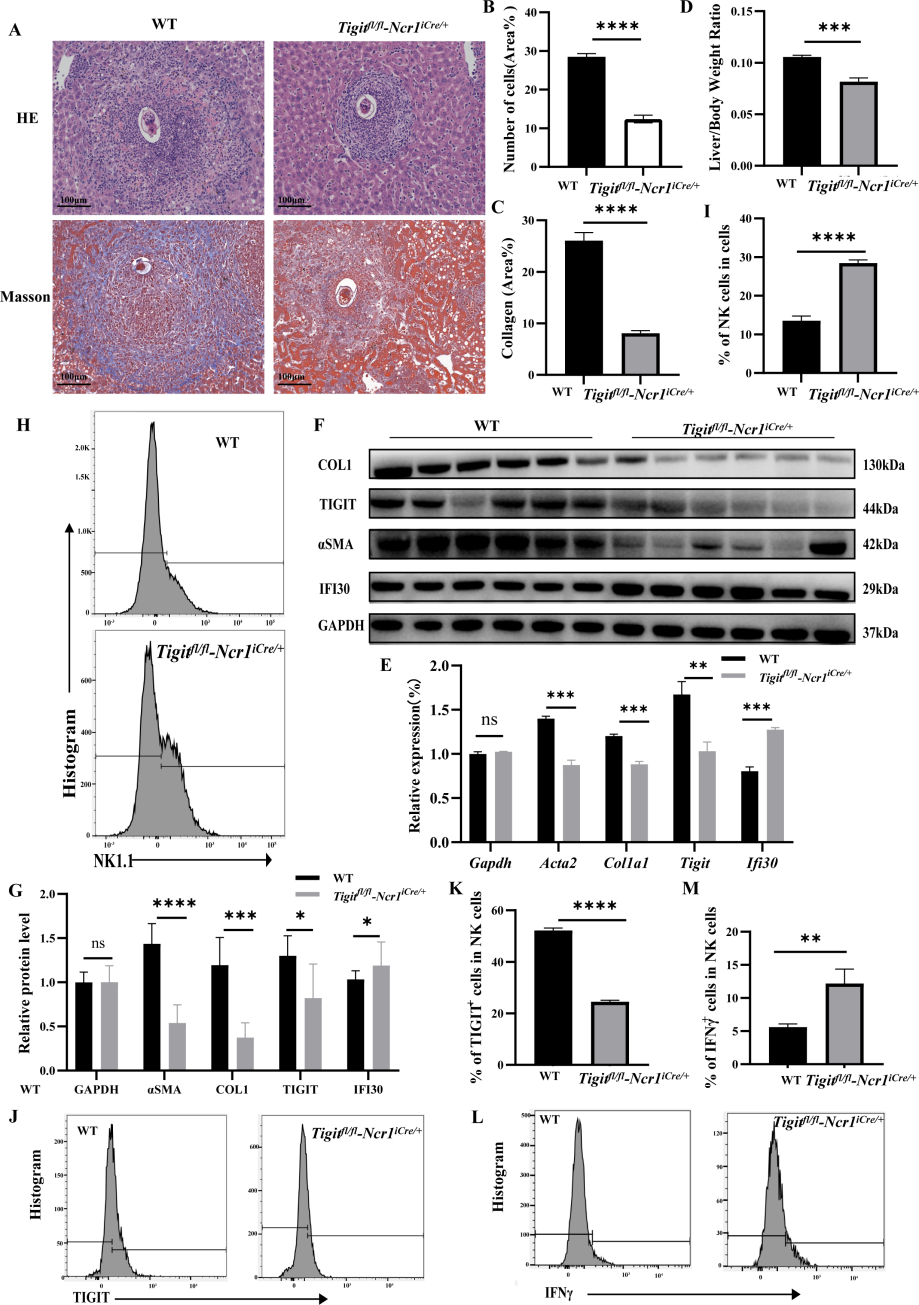
NK cell–specific *Tigit* knockout alleviates schistosomiasis-induced liver fibrosis *in vivo*. **(A)** Representative HE and Masson staining of liver tissue from WT and *Tigit*-knockout mice 6 weeks post-infection. **(B, C)** Quantification of inflammatory infiltration and collagen deposition. **(D)** Liver-to-body weight ratio in WT and *Tigit*-knockout mice. **(E)** RT-qPCR analysis of *Acta2*, *Col1a1*, *Fn*, and *Tigit* mRNA expression in hepatic tissue. **(F, G)** WB detection and quantification of α-SMA, COL1, TIGIT, and IFI30 protein levels. **(H, I)** Flow cytometry and quantification showing increased NK cell proportions in *Tigit*-knockout mice. **(J, K)** Frequency of TIGIT^+^ NK cells. **(L, M)** Proportions of IFNγ^+^ and NK cells, demonstrating enhanced NK cell activation following *Tigit* knockout. *P < 0.05, **P < 0.01, ***P < 0.001, ****P < 0.0001.

To determine the *in vivo* role of TIGIT in schistosomiasis-induced liver fibrosis, NK cell–specific *Tigit*-knockout mice were generated using gene-editing technology and subsequently infected with *S. japonicum*. Hepatic egg burden was first quantified to assess infection intensity. No significant difference in liver egg counts was observed between wild-type (WT) and *Tigit* knockout mice, indicating that the differences observed in subsequent pathological and immunological outcomes were not attributable to variations in parasite burden (**Supplementary Figure 2C**). Six weeks after *S. japonicum* infection, liver fibrosis was evaluated in both WT and *Tigit*-knockout mice by HE and Masson staining (**Figure 4A**). Compared with WT mice, *Tigit*-knockout mice exhibited markedly reduced inflammatory cell infiltration on HE staining (**Figure 4B**) and a notable decrease in collagen fiber deposition on Masson staining (**Figure 4C**). Consistently, the liver-to-body weight ratio was significantly lower in *Tigit*-knockout mice than in WT controls at 6 weeks post-infection (**Figure 4D**).

Molecular analyses supported these histological findings. Both RT-qPCR (**Figure 4E**) and WB (**Figures 4F, G**) demonstrated that the hepatic expression levels of *Acta2*, *Col1a1*, *Fn*, and *Tigit* were significantly decreased in *Tigit*-knockout mice compared with WT counterparts. Flow cytometric analysis of hepatic nonparenchymal cells isolated 6 weeks after infection revealed an increased proportion of NK cells (**Figures 4H, I**) and a significant reduction in TIGIT^+^ NK cells (**Figures 4J, K**) in *Tigit*-knockout mice. Moreover, IFNγ secretion by NK cells was markedly enhanced following *Tigit* deletion (**Figures 4L, M**). These results indicate that NK cell–specific *Tigit* knockout effectively alleviates schistosomiasis-induced liver fibrosis by restoring NK cell abundance and functional activity.

## Discussion

Schistosomiasis is a prevalent parasitic disease in which liver fibrosis represents the principal pathological manifestation and remains the leading cause of mortality among affected patients (25, 26). Dysregulated host immune responses are key drivers of disease progression during chronic infection (27, 28). Previous studies have demonstrated that upregulation of the inhibitory receptor TIGIT on NK cells is a critical factor contributing to their functional exhaustion (29). However, the precise molecular mechanism underlying this process has not been fully elucidated (30). The present study investigated the role of TIGIT in modulating NK cell function and its contribution to schistosomiasis-associated liver fibrosis. Our findings provide new insights into the immunoregulatory mechanisms linking NK cell dysfunction with fibrogenesis and identify TIGIT as a potential therapeutic target for the treatment of schistosomiasis-induced liver fibrosis.

This study provides preliminary evidence elucidating the molecular mechanism by which TIGIT regulates NK cell function and contributes to liver fibrosis. In the mouse model of *S. japonicum* infection, we observed that TIGIT expression on NK cells progressively increased with infection duration, peaking at 4–6 weeks post-infection. This period coincided with a marked rise in liver fibrosis indicators, suggesting that TIGIT participates in fibrogenesis by suppressing NK cell activity. Concurrently, NK cells exhibited clear signs of functional exhaustion. These findings align with previous observations in chronic viral infections and tumor microenvironments, where TIGIT^+^TIM-3^+^ double-positive NK cells in patients with HBV-associated hepatocellular carcinoma displayed a profoundly exhausted phenotype characterized by impaired cytotoxicity, diminished cytokine production, and reduced proliferative capacity (28, 30, 31). Similarly, in acute myeloid leukemia, TIGIT^+^ NK cell subsets exhibit immunosuppressive characteristics, and *TIGIT* blockade significantly enhances the cytotoxic activity of NK92 cells against AML cells (32).

As an immune checkpoint molecule, TIGIT contributes to disease progression primarily by inhibiting NK cell effector functions. IFNγ, TNF-α, granzyme B, and perforin are the principal cytotoxic mediators of NK cells (33–35), and reduced secretion of these molecules diminishes NK-mediated killing of HSCs (15, 36, 37). Consistent with this, *TIGIT*-knockdown NK92 cells demonstrated significantly increased IFNγ expression and enhanced cytolytic activity against LX-2 hepatic stellate cells, indicating that TIGIT modulates NK cell function largely through IFNγ-dependent mechanisms.

Transcriptomic analysis further revealed that *TIGIT* knockdown markedly upregulated *Ifi30* expression and enhanced NK cell proliferation, as evidenced by an increased proportion of cells in the S phase. *Ifi30* is an IFNγ-inducible gene (38, 39), and prior studies have shown that its translation is repressed by inhibitory elements within the 5′ and 3′ untranslated regions under basal conditions. Upon IFNγ stimulation, translation inhibitors at the 3′ region are released, allowing activators to bind and initiate *Ifi30* translation (40, 41). Functionally, IFI30 promotes cell proliferation; in zebrafish embryos, *Ifi30* knockdown significantly reduces endothelial cell proliferation and migration (42). IFI30 is also highly expressed in breast cancer, where it correlates with poor prognosis, and its silencing inhibits tumor cell proliferation, migration, and invasion (43). Moreover, IFI30^+^ M1-type tumor-associated macrophages enhance immune activation by promoting CD8^+^ T-cell responses in the tumor microenvironment (44). In our study, treatment of NK92 cells with an IFNγ antagonist reversed the increase in Ifi30 expression and was accompanied by reduced BCL2 and elevated BAX levels, confirming that the IFNγ–IFI30 axis plays a pivotal role in regulating NK cell proliferation and apoptosis.

Finally, in the NK-specific *Tigit*-knockout mouse model, *Tigit* deficiency significantly attenuated schistosomiasis-induced liver fibrosis, as evidenced by reduced inflammatory infiltration, decreased collagen deposition, and lower expression of fibrotic markers in liver tissue. These *in vivo* findings provide direct evidence for the critical role of TIGIT in promoting schistosomiasis-associated liver fibrosis through suppression of NK cell function.

**Figure 5:**
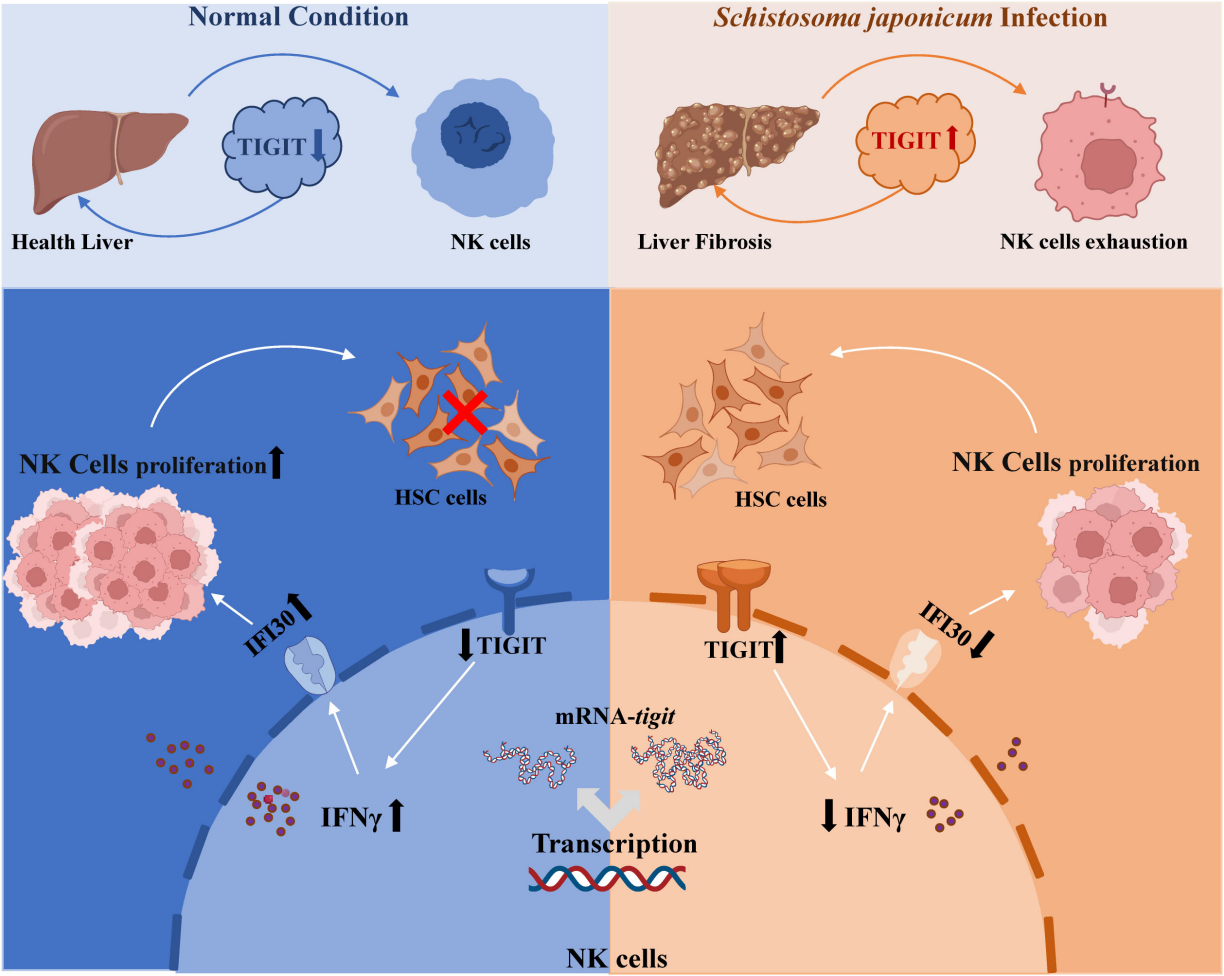
Proposed working model of the TIGIT–IFNγ–IFI30 signaling axis in schistosomiasis-induced liver fibrosis. Schematic illustration showing that upregulation of TIGIT on NK cells during *S. japonicum* infection suppresses IFNγ secretion and downregulates *IFI30* expression, leading to NK cell exhaustion and reduced cytotoxicity toward hepatic stellate cells. Conversely, TIGIT knockout restores IFNγ–IFI30 signaling, promotes NK cell proliferation and function, and mitigates hepatic fibrosis progression.

This study revealed the critical role of the TIGIT–IFNγ–IFI30 signaling axis in regulating NK cell function and schistosomiasis-induced liver fibrosis through both *in vivo* and *in vitro* experiments (**Figure 5**), underscoring its theoretical and translational significance. We elucidated the mechanism by which TIGIT acts as a negative regulator of NK cell activity and identified IFI30 as a pivotal intermediary molecule within this pathway, providing novel insight into the immune regulatory mechanisms governing NK cell dysfunction during chronic infection. These findings contribute to a deeper understanding of the immunopathogenesis of schistosomiasis-associated liver fibrosis and lay a theoretical foundation for the development of TIGIT-targeted immunotherapeutic strategies.

Despite these advances, certain limitations remain. Although IFI30 was shown to play an important role in TIGIT-mediated regulation of NK cell proliferation, the precise molecular mechanisms and downstream signaling pathways require further investigation. Moreover, as this study was primarily based on a mouse model, additional validation using clinical samples will be essential to confirm the relevance of TIGIT as a biomarker and potential therapeutic target in human schistosomiasis. These considerations highlight important directions for future research aimed at fully elucidating the role of TIGIT in the immune regulation of schistosomiasis-induced liver fibrosis.

## Data Availability

The original contributions presented in the study are included in the article/**Supplementary Material**. Further inquiries can be directed to the corresponding authors. RNA-seq raw data have been deposited in the Genome Sequence Archive (GSA) database of the China National Bioinformation Center (https://ngdc.cncb.ac.cn/gsa/) with the number CRA037766.
