# Supplementary figures and images for: TIGIT impairs NK cell antifibrotic activity through the IFNγ–IFI30 axis in schistosomiasis-induced liver fibrosis
Source: Front Immunol. 2026 Feb 19;17:1766930. doi: 10.3389/fimmu.2026.1766930 (PMC12960536; doi:10.3389/fimmu.2026.1766930)

Figure3D

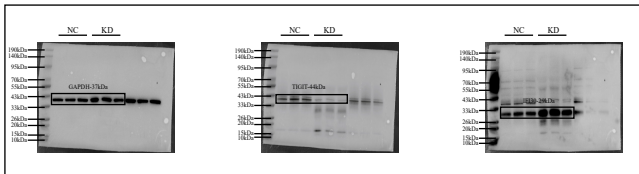

Figure3I

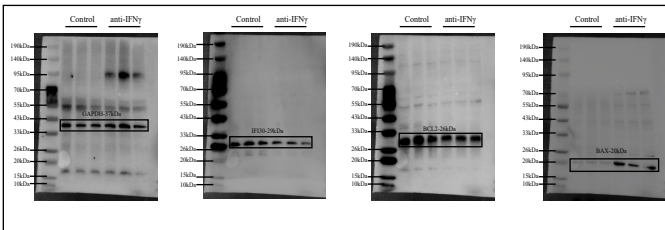

Figure4F

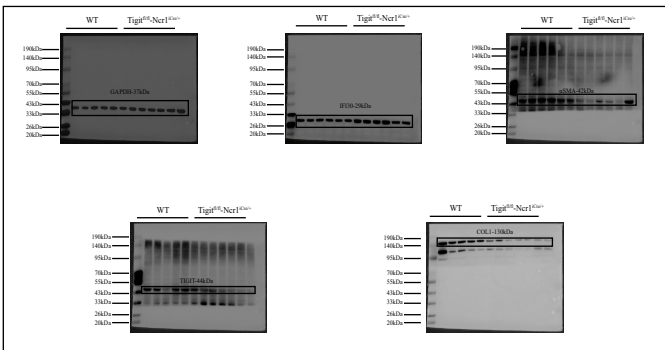

Supplement: Supplementary file 1 [file DataSheet1.pdf]

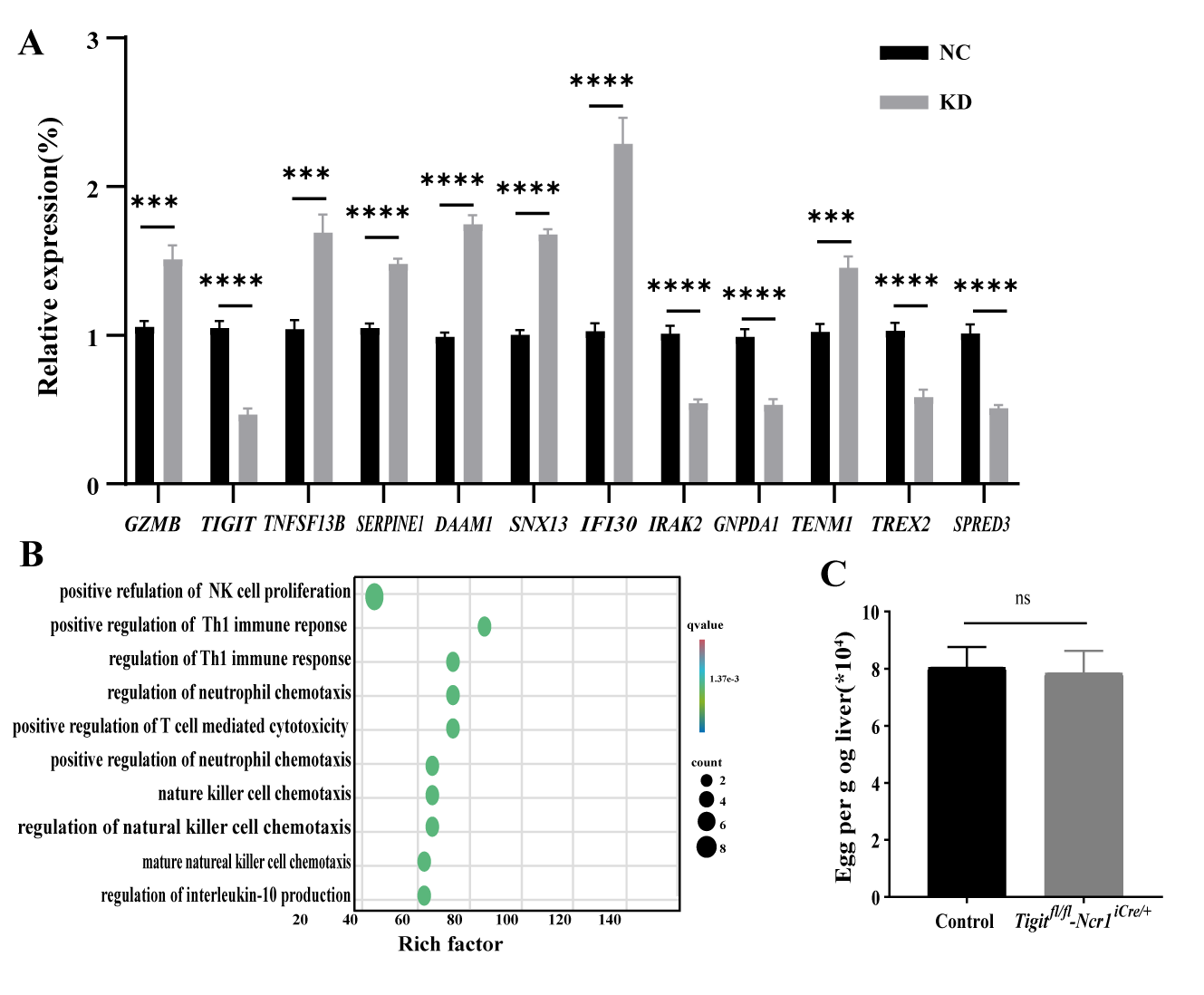

Supplement: Supplementary file 2 [file Image1.tif]
